# Is host-schistosome coevolution going anywhere?

**DOI:** 10.1186/1471-2148-7-91

**Published:** 2007-06-13

**Authors:** Joanne P Webster, Jaya Shrivastava, Paul J Johnson, Lynsey Blair

**Affiliations:** 1 Department of Zoology, University of Oxford, South Parks Road, Oxford OX1 3SY, UK.; 2 Department of Infectious Disease Epidemiology, Faculty of Medicine, Imperial College, Norfolk Place, London, W2 1PG. UK.

## Abstract

**Background:**

That pathogens and hosts coevolve is a powerful concept with broad theoretical and applied implications spanning from genetic theory to the medical and veterinary sciences, particularly in the context of infectious disease epidemiology. A substantial body of theory has been developed to explore the likelihood and consequences of coevolution, but few empirical studies have been conducted to test these theories, particularly for indirectly-transmitted pathogen-host systems. We initiated replicate longitudinal host-schistosome co-selection trials under different host genotype combinations: *Schistosoma mansoni *parasite lines were co-selected with populations of either previously resistant-selected *Biomphalaria glabrata *host genotypes, or unselected susceptible *B. glabrata *genotypes, or a mixed population of the two. All parasite lines were also passaged through their obligatory mammalian definitive host at each generation.

**Results:**

We demonstrated variation in, and a reciprocal impact on, the fitness of both host and pathogen phenotype and genotype, an outcome dependent on the combinations of genotypes involved, and evidence of change over time. Most apparent was the observation that parasites appeared to rapidly adapt to those intermediate hosts previously selected for resistance.

**Conclusion:**

Our results illustrate the potential for host-schistosome coevolution and, in particular, suggest that host resistance may be a temporary phenomenon in nature due, in part, to rapid counter-adaptations by parasites.

## Background

Host-parasite coevolution is driven by the reciprocal evolution of host resistance [1] and parasite infectivity and/or virulence [2] (see Appendix for definitions). Coevolution maintains polymorphisms at relevant loci [3,4] and has important repercussions for traits such as host-parasite compatibility, range and virulence [5]. Understanding how pathogens respond to evolved changes in host characteristics may also provide a good model for their all too apparent potential to respond to other kinds of selective pressures, such as the use of new drugs or vaccines to combat disease [6,7]. A substantial body of theory has been developed to explore the likelihood and consequences of coevolution. Classic theories, such as that of the Red Queen Hypothesis [8-11], provide a conceptual underpinning to discussions of biological evolutionary arms races or reciprocal change. However, direct tests of coevolution remain elusive and empirical evidence of host-parasite coevolution is rarely available, particularly for vertebrate host-parasite populations [12]. Some have taken this to suggest that either host-pathogen coevolution does not occur or that it is not important [13,14]. Others, in contrast, have suggested that in order to detect coevolution we must improve our techniques for how to look for it [12].

Host-parasite coevolution requires additive genetic variation in the relevant host and parasite traits, reciprocal effects on the fitness traits of the two populations, and a dependence of the outcome of the host-parasite interaction on the combinations of genotypes involved. Whilst evidence for each is required to demonstrate the potential for coevolution, demonstrating that coevolution is actually occurring requires evidence of change, whether directional or non-directional, in both host and parasite [12]. Coevolution may be expected to be most apparent where a specialist parasite exerts a strong selection pressure on its host as well as *vice versa*, where the relevant host and parasite traits have high heritability, and where parasite and host generation times are short. Many of these requirements are met in *Schistosoma *spp. (Platyhelminthes; Trematoda), at least within the snail-schistosome-rodent system, and hence may provide us with a useful model for examining potential coevolutionary interactions and thereby an insight into the genetics of adaptation in general. Schistosomes are the causative agent of schistosomiasis, a macroparasitic disease of profound medical and veterinary importance, with some 600 million people exposed and 200 million infected at any time throughout the tropical world [15]*. Schistosoma *spp. have an indirect lifecycle involving obligatory alternation of generations between mammalian definitive and molluscan intermediate hosts. Transmission between hosts occurs via free-swimming larval stages, miracidia (infective to the mollusc) and cercariae (infective to the mammal). Prevalence and transmission of schistosome infections in natural populations is highly variable across space and time [16,17]. Although the precise genetic or molecular mechanisms have not yet been identified, snail resistance and susceptibility, and schistosome virulence and infectivity, have each been demonstrated to have heritable, strain-specific bases [16,18], and variability for each trait maintained through a range of cost-benefit trade-offs [16,19].

The aim of the current study was to initiate, to our knowledge, the first experimental co-selection investigation to determine the potential for coevolution in an indirectly-transmitted animal macroparasite system [12]. In order to test whether snails and their schistosomes have the potential to coevolve we designed a longitudinal co-selection laboratory study under a combination of differing host genotype pressures. Replicate lines of *Biomphalaria glabrata *were set up consisting of snails either previously artificially-selected over multiple generations for increased resistance to *Schistosoma mansoni *infection (R-lines), or unselected susceptible lines (U-lines), or mixed 'populations' of both resistant-selected and unselected host genotypes together (R:U-lines). Due to the higher selective pressure imposed upon parasites passaged through the already resistant-selected host lines (R), such parasites may be predicted to demonstrate faster reciprocal change or adaptation than those passaged through the initially unselected hosts (U). In contrast, in terms of the hosts, we may predict faster change within the unselected (U) snails than in their previously resistant-selected (R) counterparts. As natural snail meta-populations show heterogeneity in resistant and susceptible genotypes [17], and because theoretical predictions have suggested that it may be slower for parasites to evolve counter-adaptations as heterogeneity in host resistance is enhanced [20], the mixed (R:U) line was set up here to present a potentially more epidemiologically and evolutionary realistic experimental simulation.

Uniquely amongst selection studies [4,6,21,22], the current design also encompassed more than one host species, where all schistosomes were exposed to the contrasting pressures of both their co-selected invertebrate intermediate host species and their obligatory mammalian (mouse) definitive host species at each generation. This is important because having a life cycle involving two or more obligatory host species has been predicted to constrain a parasite's ability to coevolve with either host [23,24]. Alternatively, as schistosomes have been demonstrated to display potential fitness compensatory responses through differential between-host-species transmission and establishment dynamics [23,25], one may predict any observed changes in parasite infectivity, or virulence, to the co-selected intermediate host here may also influence these traits at the definitive host stage. Therefore, despite the inherently increased logistical demands and potential to decrease the likelihood of clearly observing coevolution [23,24], inclusion of a definitive host stage within our experimental design further helps ensure the data gathered are biologically realistic and generalizable to the natural situation.

A further quality unique to the current study was that the reciprocal impact of host and parasite was investigated from both a phenotypic and genotypic perspective. Microsatellite DNA has a high mutation rate, between 10^-4 ^to 5 × 10^-6^, thus mutational changes can be detected over a short time period, making them, together with their neutrality, co-dominant expression and allelism, an ideal molecular tool for such studies. If host and parasite genotype have a reciprocal impact upon each other, we may predict that these neutral molecular markers would reveal patterns of population  differentiation inparasitesand/or their co-selected hosts over generation, consistent with that observed at the phenotype level. Furthermore, even within generation one may predict differences in allele frequencies between lines. This may be expected to be most apparent within the parasite, rather than host, lines here due to differential success in penetrating and establishing within different host genotypes, according to their resistance status, in addition to any subsequent mutation [26,27].

Finally, the current study also incorporated cross-infections between selected parasite lines with novel host lines at each generation, aimed to disentangle any potential change in parasite characteristics independent of host factors and/or to detect the strain-specificity of any response [16]. For the former, one may predict any phenotype, such as a change in infection rates in co-selected snails over time, if displayed also in novel snails exposed to the same parasite, would indicate a predominantly parasite-focused trait change. In contrast, a lack of associated phenotype in novel snails may indicate a predominantly host-focused change amongst the co-selected snails. However, any strain-specificity inherent in this system may equally be likely to reduce or mask any such host-parasite differentiation. Strain specificity, where any phenotypic trait is displayed only within the host-parasite co-selected combination, is a fundamental assumption of many coevolutionary models and theories, including that of frequency dependent or Red-Queen coevolution [8-11], and may account for the greater compatibility amongst many sympatric snail-schistosome populations in the field [28]. Such strain-specificity, where selected snails were exposed to novel parasites and/or selected parasites were exposed to novel snails, has been documented within previous experimental studies on each of host resistance and susceptibility and parasite infection intensity, infectivity and virulence traits in this snail-schistosome system [16,18,29]. Therefore whilst cross-infection experiments serve as an important complement to the major co-selection experimental design, alone, due to any inherent strain-specificity, they would not be sufficient to test for the potential for host-parasite coevolution in this system.

Our study is thus believed to represent one of the first experimental co-selection investigations in an animal-parasite system, specifically that for an indirectly-transmitted macroparasite [12]. Through demonstration of variation in, and a reciprocal impact on, the fitness of both host and parasite phenotype and genotype, combined with evidence of change over time, most apparent within those parasites co-selected with resistant snail genotypes, the current study provides an important stepping stone in demonstrating the potential for host-schistosome counter-adaptation and coevolution.

## Results

### Within the intermediate host

**Figure 1:**
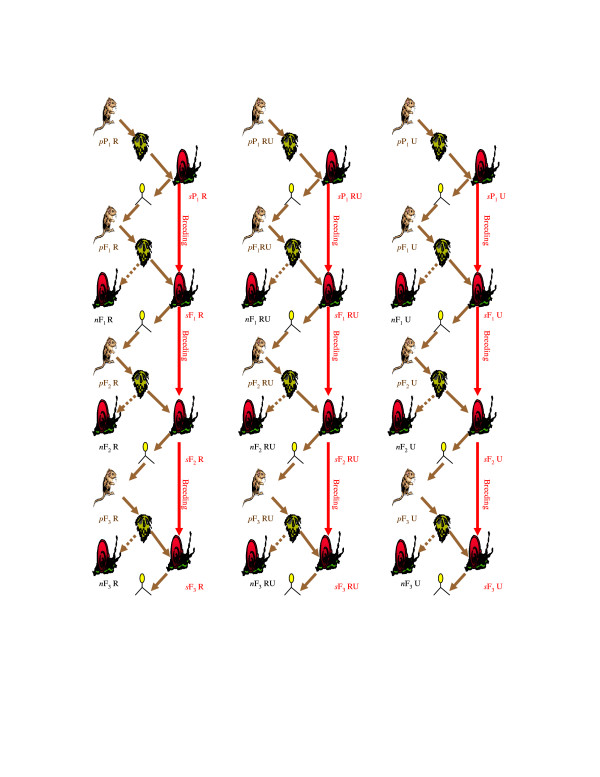
**Host-parasite co-selection (and cross-infection) design**. Solid lines represent host and parasite co-selection over generations. Only one replicate illustrated, for simplicity, from: three lines of *S. mansoni *co-selection passage through either R (artificially-selected resistant snails); R:U (equal mix of resistant-selected and unselected snails) or U (unselected snails), of four replicates per co-selection line per P1-F3generation (*s *for snail; *p *for parasite line) mass exposed to a matched dose of *S. mansoni*, at n = 20 *s *snails per 'population' per line replicate per generation. Broken lines represent parasite (*p*) exposure to novel (*n*) non-coselected snail (cross-infections) at n = 10 *n *snails per 'population' per line replicate per generation). Given the generation times of the parasite for harvesting in each of its obligatory host stages (7 weeks in the snail; 8 weeks in the mouse), each line/replicate here represents a minimum of 62 weeks (approx 1.2 years) continuous co-selection data collection (in addition to the preceding approx 2.4 years continuous snail host only P1-F6 artificial selection for resistance).

**Figure 2:**
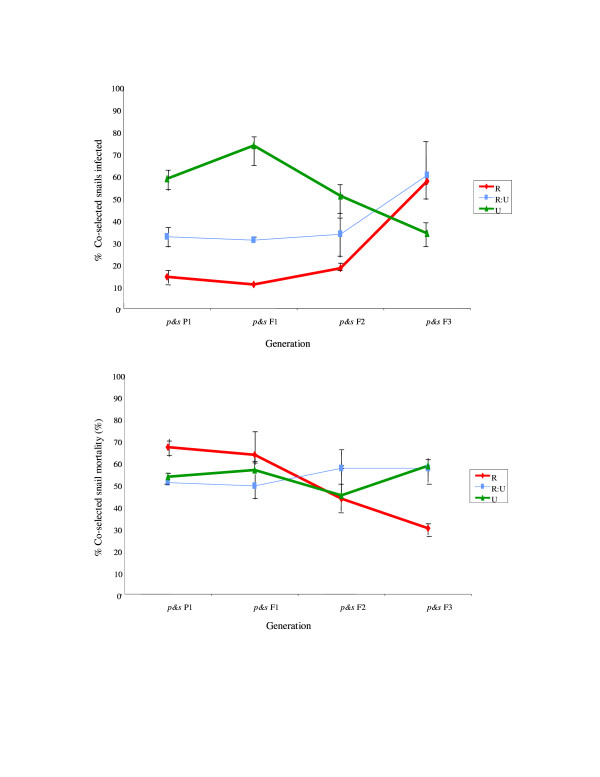
**a **Infection % prevalence (parasite infectivity and/or snail susceptibility) in the the co-selected intermediate hosts over line (*p&s *R, RU, U) and generation (*p&s *P1-F3) (pooled across replicates within line and within generation) (see Figure 1 for coding). **b. **Virulence (mean/SEM snail mortality) in the co-selected intermediate hosts over line (*p&s *R, RU, U) and generation (*p&s *P1-F3) (pooled across replicates within line and within generation) (see Figure 1 for coding).

Within the co-selection study, parasite infection rate (parasite infectivity and/or host susceptibility) was higher in the U snail lines than in their R or R:U counterparts overall (*F*_2,37 _= 9.2, *p *= 0.0006). There was, however, evidence of a significant interaction between selection line and generation (*F*_2,37 _= 3.3, *p *= 0.0003), where parasite infection rate increased significantly with generation in the R lines (*F*_1,10 _= 4.33, *p *= 0.04), and to some extent in the R:U lines, while declining non-significantly with generation for the U snail lines. In other words, whilst snail infection prevalence was significantly highest amongst U line host-parasite combinations at the start of the co-selection study (F_2,8 _= 5.23, *p *= 0.03), this trait was reversed by the final generation with highest infection prevalence observed in the R-line host-parasite combinations (Figure 2a - see fig 1 for coding).

Virulence, in terms of snail mortality, also showed a different trajectory over time between lines (line × generation interaction term: *F*_2,55 _= 2.7, *p *= 0.07), where snail mortality declined significantly with generation through the R lines (*F*_1,18 _= 5.13, *p *= 0.04), but showed no trend for the other two lines (Figure 2b).

**Figure 3:**
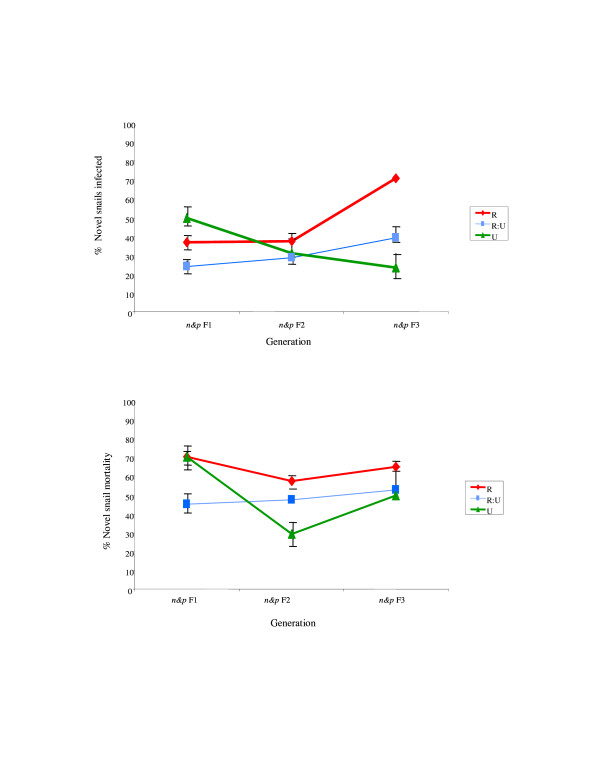
**a **Infection % prevalence (parasite infectivity) in the Novel (i.e. not co-selected) intermediate hosts over line (*p *R, RU, U through *s *N) and generation (*p *F1-F3) (pooled across replicates within line and within generation) (see Figure 1 for coding). **b **Virulence (mean/SEM snail mortality) in the Novel (i.e. not co-selected) intermediate hosts over line (*p *R, RU, U through *s *N) and generation (*p *F1-F3) (pooled across replicates within line and within generation) (see Figure 1 for coding).

Within the cross-infection study (parasite lines from the co-selection study exposed to novel control snails), a similar pattern to that within the co-selected lines was observed: there was also evidence of a significant interaction between selection line and generation (*F*_2,28 _= 5.31, *p *= 0.01), where parasite infection rate increased with generation in the R lines, and to some extent in the R:U lines, while declining with generation for the U snail lines (Figure 3a). There was, however, no significant difference between lines over time in terms of parasite virulence to these novel snails (Figure 3b).

### Within the definitive host

**Figure 4:**
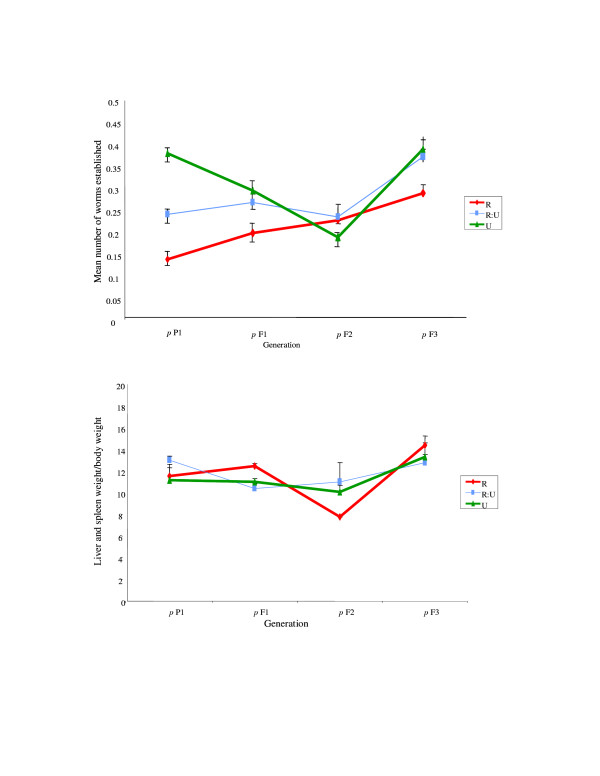
**a **Parasite infectivity (mean/SEM number of worms established per mouse per 220 cercariae exposure) to the definitive hosts over line (*p *R, RU, U) and generation (*p *P1-F3) (pooled across replicates within line and within generation) (see Figure 1 for coding). **b **Virulence (mean/SEM weight of liver and spleen as a percentage of total body weight) to the definitive hosts over line (*p *R, RU, U) and generation (*p *P1-F3) (pooled across replicates within line and within generation) (see Figure 1 for coding).

In the definitive host, the impact of both line and generation was as apparent as that observed in the co-selected intermediate hosts. Infectivity tended to increase through the generations (*F*_1,36 _= 3.91, *p *= 0.06) and, although there was no statistical evidence for a line × generation interaction, the slope was steepest for those parasites passaged through R snails (Figure 4a). Overall, infectivity to the mouse was also significantly lower in parasites passaged through the R snail lines than from either R:U or U snails (the mean proportions of worms establishing in mice across all generations (with SEMs) were R = 0.19(0.02), RU = 0.27(0.02), U = 0.32(0.02); F_2,36 _= 5.25, *p *= 0.010).

Parasite virulence within the definitive host, in terms of hepatosplenomegaly, did not differ in response to the selection status of the intermediate host through which they had been passaged, nor did it show a trend with generation (Figure 4b).

### Genotypic change in host and parasite

**Figure 5:**
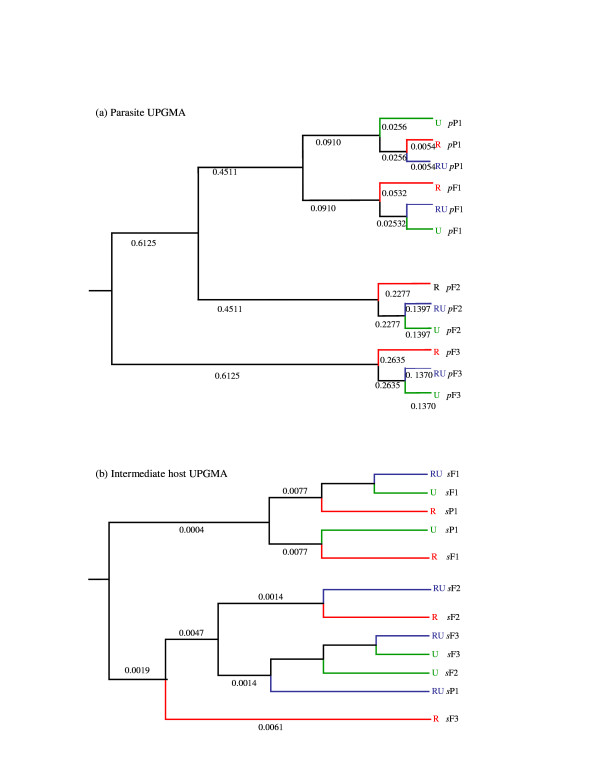
**a **Unweighted Pair Group Method with Arithmetic Mean (UPGMA) phenogram generated using co-ancestry identity for parasites over line (*p *R, RU, U) and generation (*p *P1-F3) (pooled across replicates within line and within generation) (see Figure 1 for coding). **b **Unweighted Pair Group Method with Arithmetic Mean (UPGMA) phenogram generated using co-ancestry identity for intermediate hosts over line (*s *R, RU, U) and generation (*s *P1-F3) (pooled across replicates within line and within generation) (see Figure 1 for coding).

Molecular analyses also provided support for an impact of host on parasite genotype and, to some extent, *vice versa*. The Unweighted Pair Group Method with Arithmetic Mean (UPGMA) phenogram for parasites generated using co-ancestry identity revealed clustering based on generation and on line (Figure 5a). Indeed, within each of the increasing co-selected F1-F3 generations, the parasites co-selected through the R snail lines consistently clustered separately from the R:U and U-lines, with the F3 generation being most different, suggesting an impact of selection rather than random genetic differentiation. As for the intermediate hosts, whilst there was no clear clustering pattern between lines by generation, within each generation the R snail lines also tended to cluster separately from the R:U and U-lines, particularly by the F3 generation (Figure 5b). Comparison of Fst estimates between the first and final generations for both the intermediate host and parasite lines revealed greater genetic variation within parasite (P1:RF3-0.74; P1:RUF3-0.63; P1:UF3-0.65) than within the intermediate host (P1:RF3, P1:RUF3; P1:UF3-all values <0.001). Indeed, all the Fst values obtained here were significant (P < 0.01) suggesting great differentiation within parasites lines based on generation compared to the intermediate host (very low differentiation). Random genetic drift alone could not explain such consistent differences between co-selection lines and/or the coherent association between the genotype data with that of the phenotypic traits observed as demonstrated in this study.

## Discussion

The results presented here provide empirical support to suggest the potential for host-schistosome coevolution, and in particular that consistent with Red Queen coevolution [8-11] where hosts must continually evolve in order to achieve transient resistance to coevolving parasites tracking them. Complementary to the previously published host-only selection experiments, which demonstrated that *B. glabrata *genotypes resistant or susceptible to *S. mansoni *can increase in frequency in response to just one or two generations of selection [16,18,30], the current host-parasite co-selection experiments demonstrate a subsequent reciprocal change in both host and parasite populations. Further indication of evolution in host resistance may be suggested here as the decrease in infection prevalence over generation, and hence potential increased resistance to infection, within the originally unselected (U) snail lines (Figure 2a). Indication that *S. mansoni *can rapidly counter-adapt to such differential host genotype pressures was observed here as the phenotypic and genotypic changes evident in parasite lines, expressed to both intermediate and definitive host, co-selected with the originally resistant-selected (R) snails (Figures 2, 3, 4, 5). Such rapid counter-adaptations, in addition to the previously documented strain-specificity [16,18] and high costs of resistance inherent in this system [31], are thereby consistent with a transient nature and lack of fixation of resistant genotypes in natural populations [8,11,12,31,32]. Indeed, over a greater number of co-selected generations, one may well predict cycling between host resistance and parasite infectivity in this system.

Further potential for polymorphism in natural populations may be suggested here by the corresponding decrease in virulence observed amongst R-lines as infectivity increased over generation (Figures 2a &2b). Such patterns may be explicable, in part, by a relatively higher individual infection dose and subsequent intra-host parasite competition within those comparatively few infected P1 R-line snails. High parasite exposure doses and intra-host competition levels are both factors previously demonstrated to be associated with high virulence to these snails [16,33,34]. As parasite infectivity increased and/or snail resistance decreased in subsequent generations within these R-lines, a relatively greater proportion of snails would be infected at a presumably correspondingly lower individual parasite dose (as the total mass miracidial exposure dose per line remained constant over time), and hence intra-host competition and subsequent virulence would be reduced [35]. An alternative, not mutually exclusive explanation, may relate to the high costs to the host inherent within their immune defence, where a lack of mounting such a response would result in the observed increasing parasite establishment and declining host virulence over time amongst these R-lines [36]. However, the lack of any consistent corresponding change in virulence over time amongst the U-lines (Figure 2b &3b), despite an apparent change in parasite infectivity (Figures 2a &3a), may suggest other factors too may be involved in this host-parasite relationship. This latter may also provide further evidence to suggest that infectivity does not appear to equal virulence in this snail-schistosome system [16,25], although it remains to be ascertained as to which trait may have the greater genetic variability. It should be acknowledged, nevertheless, that the potential strength of selective pressure on evolution of virulence in the current experimental design may by constrained to a minimum, in contrast to the selective pressures imposed here on parasite infectivity and snail host resistance/susceptibility. Parasite exposure dose was relatively low in order to ensure sufficient snails survived to breed the next co-selected generations. Intra-host parasite competition, as mentioned above, would be subsequently minimized in most snail lines. Likewise, parasite exposure dose together with infection duration was also kept to a tightly controlled minimum within the definitive hosts, and all rodents were euthansed before parasite-induced morbidity/virulence should have developed. Such protocols were performed for ethical reasons. If higher parasite doses for intermediate and definitive hosts, and/or longer durations of infection in the latter, one may well predict stronger selective pressure on parasite virulence imposed and subsequently displayed here between lines and generations.

Of course there are many more complexities both host and parasite will be exposed to under natural settings, including constraints imposed by simultaneous interactions with multiple hosts or multiple parasite species and strains. Indeed, theoretical predictions have suggested that it may be much more difficult and slower for parasites to evolve counter-adaptations as heterogeneity in host resistance is enhanced [20], and in accordance with this the reciprocal impact appeared reduced within the mixed R:U lines in the current study (Figures 2a &2b). Yet this is not to suggest that such adaptation and counter-adaptation cannot occur, even under highly heterogeneous natural conditions. Furthermore, increased heterogeneity has also been proposed to increase the likelihood for coevolution in some cases, where for example, host defence against genetically heterogeneous parasite infections may require more resources to be effective and thus may result in increased pressure for the evolution of host resistance and/or parasite infectivity [37-39]. Likewise, whilst having a life cycle involving two or more obligatory host species has been predicted to constrain a parasite's ability to coevolve with either host [23,24], one could conversely suspect it may be less costly for the parasite to adapt towards the definitive host here, as this represents the more constant host genotype, in contrast to the potential reciprocally changing intermediate host. The apparent increase in parasite infectivity to the intermediate host with generation in the R-line (Figure 2a and 3a) did appear to be reflected here in the definitive host stage (Figure 4a). It is plausible that a similar scenario may occur within natural host-schistosome interactions, particularly considering the longer generations of the definitive host, either human or rodent, relative to the snail.

Such elucidation of the evolutionary dynamics of such schistosomes and their hosts holds considerable applied significance for their control and management. For example, one theoretically proposed snail-mediated control strategy, the genetic control theory (GCT) for schistosomiasis, contends that it is possible to reduce the size of schistosome populations by genetic manipulation of the snail intermediate host [40-42]. The technique advocates collection of resistant snails from natural populations, artificially selecting for resistance within the laboratory and returning descendants to the site of disease transmission. The theory rests on the central assumptions that resistant snails will have an evolutionary fitness advantage over their susceptible-infected counterparts, thus spreading the genes for resistance through the population, and that the resultant genetic perturbation due to the increase in resistant snails will be too great for the schistosome population to adjust co-evolutionarily [40]. In contrast, the apparently rapid counter-response in terms of increased parasite infectivity to, and/or increased susceptibility of, those previously resistant-selected snails observed here (Figures 2a &3a), combined with the previously documented strain-specificity and high costs of resistance inherent in this system [16,18], would appear to mitigate against the predicted success of such a control measure. Likewise, one may suspect that the essentially transient and strain-specific nature of the virulence traits observed here could constrain any potential likelihood of manipulation of schistosome virulence for public health ends [43].

There does remain, nevertheless, a need to develop and apply new, more powerful tools and methodologies, in particular those aimed to identify reciprocal polymorphisms in genes involved in the host-pathogen interaction, for both host-schistosomes and many other theoretically and clinically important host-pathogen systems. Not surprisingly perhaps, where considerable progress has been made in these areas, convincing evidence of potential host-pathogen coevolution and counter-adaptation is beginning to appear. For instance, reciprocal molecular polymorphisms between the human Major Histocompatibility Complex (MHC) and malaria CS genes in West African populations have been identified and the co-distributions of pathogen and host genotypes are consistent with selection pressures exerted on each other [44]. Likewise, one explanation for the reduced virulence of simian immunodeficiency virus (SIV) in sooty manabeys is that this and closely related species have evolved mechanisms to silence the CCR5 receptor [45]. Suggestive of a coevolutionary arms race, there is then evidence of subsequent SIV counter-adaptation to CCR5 inhibition, where red-capped mangabeys harbour SIV strains that utilize CCR2 instead of CCR5 [46,47]. Recent research may even suggest that the human immune response is also already driving adaptation of the human immunodeficiency virus (HIV) and, at the same time, the virus is driving evolution of human immune genes [48]. Researchers have observed that, in a small proportion of infected people, the virus is successfully controlled by the immune system. It appears that the cytotoxic T lymphocytes (CTLs) principally involved in killing HIV-infected cells are recognising fragments of viral proteins presented on HLA-B molecules, and that these are the immune responses against which HIV is adapting the fastest. Similarly, the success of the immune response in controlling HIV infection, and therefore the speed of progression to AIDS, is primarily determined by the particular HLA-B genes expressed by each individual. Such data thereby suggest an explanation for the more rapid evolution of HLA-B. We anticipate that, as further analytical tools are developed, further evidence indicative of host-pathogen coevolution will appear across a whole range of systems, even for comparably long lived and complicated host-parasite interactions such as that of the indirectly-transmitted macroparasite system examined here. Moreover, we suspect that the subset of host genes involved in coevolutionary interactions will turn out to be of particular biomedical importance, since they are likely to be major determinants of susceptibility, infectivity and virulence and should provide insights not only into the ways that host and pathogen adapt and counter-adapt but, perhaps more importantly, into the constraints which prevent either 'winning' the evolutionary battle outright.

## Conclusion

To conclude, the results obtained here do, at least under the tightly controlled constraints of this experimental setting, demonstrate the potential for host-schistosome coevolution, even within as little as 3–4 generations. At the same time, the current study serves to highlight both the complexities and considerations inherent in any attempt to achieve empirical evidence of host-parasite coevolution in action, particularly in complex animal-parasite systems [12]. Yet, an understanding of the evolutionary dynamics of such parasites holds considerable significance for their control and management, and this study should, at very least, help motivate further experimental and theoretical investigation of coevolution in this fascinating indirectly-transmitted system and beyond. The impact of host-pathogen coevolution on host genetics, immunology and disease epidemiology is only beginning to be better understood, and an interdisciplinary approach will be imperative to progress this exciting area, especially in today's environment of emerging and novel infections.

## Methods

### Host and parasite lines

Here we focus on co-selection between the parasite *S. mansoni *and its intermediate host *B. glabrata *(the natural host within the New World and parts of Africa), with mice (CBA/CA; Harlan Olak UK, Ltd) serving as the definitive host (since both humans and rodents act as natural definitive hosts for *S. mansoni *within Africa and the New World) [17].

Artificial selection, carefully controlling against any potential inbreeding or maternal-effect bias, was used to breed *B. glabrata *snail lines that were resistant-selected, susceptible-selected, or unselected, toward *S. mansoni *infection, as has been described in detail elsewhere [18]. There were highly significant differences between snail lines in the % infection prevalence following exposure to five miracidia by the F1 generation (log-linear analysis: χ^2 ^= 93.83, d.f. = 2, *P *= 0.001) and this difference remained significant in all subsequent generations (F1 to the F6 generation used here).

### Co-selection experimental design (Figure 1)

Replicate groups of 20 adult (as determined by the onset of egg laying) size- and age-matched *B. glabrata *containing graded proportions of previously resistant-selected and unselected snail lines were placed in tanks (30 cm × 15 cm × 10 cm); four tanks contained 20 resistant-selected snails (100% Resistant 'R'), four contained 10 resistant- and 10 unselected snails (50% Resistant: Unselected 'R:U') and four tanks contained 20 unselected snails (100% Unselected 'U'). Snails for each line/tank were mass exposed to a maximum of 100 miracidial dose (mean of 5 per snail), from a previously unselected laboratory *S. mansoni *line. Using mass rather than individual exposure here allows the miracidia to 'choose' the snails to infect, which may also be more comparable to the natural situation. At week 7 post-exposure, *S. mansoni *cercariae from all infected snails within each group were harvested, pooled and used to infect four mice (CBA/CA; Harlan Olak UK, Ltd) per group replicate, at a dose of 220 cercariae, by allowing the animal's feet to paddle freely for 60 minutes in 100 ml of infected water. The mice were kept for 6–8 weeks in order to allow the schistosomes to mature to adults, and for the females to become gravid and to start producing viable eggs. Following this period, before the mice showed signs of illness, they were euthanized with an increasing concentration of carbon dioxide. The liver and spleen were removed, macerated through a sieve, and placed in 80 ml deionised water under a 100 watt light in order to stimulate hatching. For the F_1 _generation, a randomly selected group of 20 size- and age-matched offspring from each parental snail line was moved to a fresh tank and exposed to the corresponding miracidia harvested from the mice infected with the parasites obtained from the parental lines, such that each parasite line was co-selected with the snail line through which it had been passaged. The parasite dose remained the same through generations. Subsequently, cercariae shed by snails from all the F_1 _experimental groups were pooled, within co-selection line, and used to infect four more adult mice, as described above. This procedure was repeated across four generations in total (P_1_-F_3_).

In order to follow the potential genotypic changes in response to co-selection, 20 worms (10 male: 10 female), and 10 snails, were randomly selected from each line per P1-F3 generation and their population genetic structure analysed using six of the recently characterised microsatellite markers for *S. mansoni *[49] and 8 of the microsatellite markers for *B. glabrata *[50] (Table 1) . Total genomic DNA from ethanol preserved snails was extracted by using their foot tissue and a modified phenol-chloroform extraction protocol followed by purification with GeneClean II kit. Genomic DNA from individual schistosomes was extracted using phenol-chloroform extraction and purified using GeneClean II kit. PCR was performed using published primers with the 5' end of the forward primer for each locus fluorescently labelled using 6-FAM, NED or TET dye (Applied Biosystems), and amplification on a PTC-200 Thermal Cycler (MJ Research). Amplifications were performed in 40 μl reactions with 10 ng of genomic DNA, 5 pmoles of each primer, 1 unit of Taq polymerase, 4 μl of reaction buffer (10 mM Tris-HCl pH 9.0, 50 mM KCl, 0.1% Triton^®^X-100; Promega), 0.4 mM dNTPmix (Sigma) and 1.5–2.5 mM MgCl_2 _(Promega). Thermal cycling was conducted under the following conditions: 5 minutes at 94°C, followed by 35 cycles of 30 seconds at 94°C, 1 min at locus-specific annealing temperature, 1 min at 72°C, with a final extension at 72°C for 7 min. Products were diluted in N, N'- dimethyl formamide with GeneScan ^®^-500 [ROX] (ABI), electrophoresed using a ABI 377 sequencer; and sized using GENESCAN software v3.7 (PE Applied Biosystems) and Genotyper v 3.7(PE Applied Biosystems).

### Cross-infection experimental design

Cross-infections between selected parasite lines with novel host lines were performed at each selection generation, aimed to disentangle any potential change in parasite characteristics independent of host factors and/or to detect the strain-specificity of any response [16]. At each generation an additional set of replicate tanks of 10 novel size- and age-matched novel control snails (i.e. snails not co-selected with the parasite here, but those from a separate unselected laboratory stock line, referred to as Novel control (*n*) snails here) were exposed, at a matched dose, to the same parasite line (*p*) as the experimental co-selected snails.

### Life-history data

Intermediate host life-history parameters were recorded for each line replicate at weekly intervals including (i) Parasite infectivity to the snail and/or snail resistance or susceptibility to the parasite was measured as the percentage of patent snails, i.e. those shedding cercariae, within each snail group [25], and (ii) Virulence was measured as the number of breeding adults remaining from the original matched snail population in each tank after 15 weeks, i.e. snail mortality.

Definitive host life-history parameters were recorded where adult schistosomes were recovered from each mouse using a modified hepatic perfusion technique. (i) Parasite infectivity was calculated as the number of worms successfully established within the definitive host following a matched 220 cercarial exposure dose [23,35] and (ii) virulence to infected mice was measured as the weight of the liver and spleen as a percentage of total body weight, as the major pathological effect of *S. mansoni *infection in definitive hosts results from granuloma formation causing hepatosplenomegaly [51].

### Statistical analyses

Evidence of phenotypic differences in all parameters between lines and across generations (where the co-selection data were analysed separately from the cross-infection study data) was investigated using a general linear modelling procedure (GLM procedure) applied with the SAS Software v.8 (SAS Inst. 1999). Dependent variables were transformed as necessary (log, square-root or arcsine-root as appropriate) to meet the model assumptions of normality of error and homogeneity of variance. Selection line (R, R:U and U) was used as a categoric independent variable. Generation (P1-F3) was treated as a continuous variable. Intermediate host dependent variables included i) infection prevalence (parasite infectivity to/resistance in the snail) and ii) mortality (virulence). Similarly i) infectivity and ii) virulence to the mouse were used as dependent variables for the definitive host. Throughout, as there were replicates and all three lines of snail occur within each, these effects were therefore crossed (in contrast to nested, where one variable is nested within another when the levels of that first variable occur only within one level of the second variable, classically when experimental subjects are assigned randomly to one or more levels of an experimental treatment, and hence not the design applied here). Non-independence attributable to multiple measurements on different replicates was therefore dealt with by treating 'replicate' as a fixed blocking factor. Inferences about the effects of interest are therefore confined to these replicates, and not generalised to a wider population of replicates that might have been used. Although treating each of the two replicates as a subject in this way yields a design where inferences cannot be generalized to a wider population of potential replicates, this does not weaken the value of the data we present with respect to the replicates observed [52]. As our effect sizes are clearly presented, and with appropriate metrics of their precision, we have chosen not to incorporate Bonferroni correction procedures here to control type-1 error rate; such procedures are increasingly viewed as unhelpful [53-56].

### Molecular analyses

As a measure of genetic distance between populations, a matrix of co-ancestry coefficients was estimated in Arlequin 2.0 [57], which considers pair-wise distance, population size and divergence time. In order to demonstrate visually the similarity of individuals or populations, UPGMA clustering (a sequential clustering algorithm, in which local topological relationships are identified in order of similarity, and the phylogenetic tree is built in a stepwise manner) was then performed on all population samples based on these pair-wise co-ancestry data. Analysis of molecular variance (AMOVA) [58] was used to quantify intra- and inter-population variability which calculates Wright's F-statistics, e.g. Fst measures which reflect the genetic differentiation between different samples [59], suggest the following qualitative guidelines for the interpretation of Fst genetic differentiation: 0–0.05 'little'; 0.05–0.15 'moderate'; 0.15–0.25 'great'; and > 0.25 indicate 'very great' genetic differentiation. The significance of departure from 0 for Fst was tested by randomising alleles between individuals in each sample (15000 permutations) and the significance level was set at P < 0.01.

## Authors' contributions

JPW designed the experiment, partially analysed the data, and wrote the manuscript, LB conducted the experiment and collected the data, LB & JS performed the molecular analyses and PJ performed the statistical analyses. All authors' contributed to the revisions of the text. All authors' read and approved the final manuscript.

## Appendix

Definitions: **Host resistance**: the genetic, biochemical and/or physiological profiles that inhibit parasite establishment, survival and/or development within the host; **Parasite infectivity**: the infective capacity of the parasite, when applied to suitable host tissues, to produce the next infective stage; **Virulence**: parasite-induced host mortality or morbidity/reduced lifetime reproductive success.

**Table 1:** Microsatellite loci used in this study.

**Locus**	**Species**	**Repeat**	**GenBank Accession no.**	**Size range**
Bgμ8 ^[50]^	*B.glabrata*	(TG)_7_TT(TG)_10_	AF157698	107–131
Bgμ10 ^[50]^	*B.glabrata*	(CA)_11_	AF157699	93–107
Bgμ16 ^[50]^	*B.glabrata*	(TC)_24_(TATC)_6_	AF157701	124–138
μBg1 ^[50]^	*B.glabrata*	(TC)_20_	AF157703	160–200
μBg2 ^[50]^	*B.glabrata*	(GT)_20_	AF157704	248–280
BgC6 ^[60]^	*B.glabrata*	(CA)_4_TT(CA)_2_CT(CA)_5_(TACA)_2_	AF216279	302–304
BgE3 ^[60]^	*B.glabrata*	(GATA)_25_	AF216269	221–253
SCGA3 ^[49]^	*S.mansoni*	(CT)_24_	AF629514	167–207
SATA12 ^[49]^	*S.mansoni*	(TA)_19_	AI395718	303–343
CA11-1 ^[49]^	*S.mansoni*	(GA)_5_n_2_(GT)_10_	AI068336	191–231
sms6-1 ^[49]^	*S.mansoni*	(GT)_17_	AF330104	148–188
sms7-1 ^[49]^	*S.mansoni*	(CA)_17_	AF330105	164–204
sms9-1 ^[49]^	*S.mansoni*	(GT)_16_	AF330106	178–208

## References

[B1] Coustau C, Chevillion C, Ffrench-Constant R (2000). Resistance to xenobiotics and parasites: can we count the cost?. Trends in Ecology and Evolution.

[B2] Read AF (1994). The evolution of virulence. Trends Microbiol.

[B3] Rainey PB, Buckling A, Kassen R, Travisano M (2000). The emergence and maintenance of diversity: insights from bacterial populations.. Trends in Microbiology.

[B4] Toft CA, Karter AJ (1990). Parasite-host coevolution. Trends Ecol Evol.

[B5] Woolhouse MEJ, Dye C (2001). Population biology of emerging and re-emerging pathogens.. Philosophical Transactions of the Royal Society Series B.

[B6] Ebert D (1998). Experimental evolution of parasites.. Science.

[B7] Gandon S, Mackinnon MJ, Nee S, Read AF (2001). Imperfect vaccines and the evolution of pathogen virulence.. Nature.

[B8] Van Valen L (1973). A New Evolutionary Law. Evolutionary Theory.

[B9] Bell G, Maynard Smith J (1987). Short term selection for recombination among mutually antagonistic species. Nature.

[B10] Jaenike J (1978). An hypothesis to account for the maintenance of sex within populations. Evolutionary Theory.

[B11] Bell G (1982). The Masterpiece of Nature: The Evolution and Genetics of Sexuality..

[B12] Woolhouse MEJ, Webster JP, Domingo E, Charlesworth B, Levin BR (2002). Biological and biomedical implications of the coevolution of pathogens and their hosts. Nature Genetics.

[B13] Little TJ (2002). The evolutionary significance of parasitism: do parasite-driven dynamics occur ex silico?. Journal of Evolutionary Biology.

[B14] Satta Y, Ohuigin C, Takahata N, Klein J (1994). Intensity of natural selection at the major histocompatibility complex loci.. Proceedings of the National Academy of Science, USA.

[B15] Chitsulo L, Engels D, Montresor A, Savioli L (2000). The global status of schistosomiasis and its control.. Acta Tropica.

[B16] Webster JP, Davies CM (2001). Coevolution and Compatibility in the Snail-Schistosome system.. Parasitology.

[B17] Wilkins HA, Rollinson D and Simpson AJG (1987). The epidemiology of schistosome infections in man.. The Biology of Schistosomes : from genes to Latrines.

[B18] Webster JP, Woolhouse MEJ (1998). Selection and strain specificity of compatibility between snail intermediate hosts and their parasitic schistosomes. Evolution.

[B19] Webster JP, Hoffman J, Berdoy MEL (2003). Parasite resistance and mate choice - battle of the genders in a simultaneous hermaphrodite.. Proceedings of the Royal Society (London) Series B.

[B20] Anderson RM, May RM (1982). Coevolution of hosts and parasites. Parasitol.

[B21] Lambrechts L, Fellous S, Koella JC (2006). Coevolutionary interactions between host and parasite genotypes. Trends in Parasitology.

[B22] Bull JJ, Badgett MR, Rokyta D, Molineux IJ (2003). Experimental evolution yields hundreds of mutations in a functional viral genome. Journal of Molecular Evolution.

[B23] Gower CM, Webster JP (2004). Fitness of indirectly-transmitted pathogens: restraint and constraint.. Evolution.

[B24] Weaver SC, Brault AC, Kang WL, J.J. H (1999). Genetic and fitness changes accompanying adaptation of an arbovirus to vertebrate and invertebrate cells. Journal of Virology.

[B25] Davies CM, Webster JP, Woolhouse MEJ (2001). Trade-offs in the evolution of virulence of schistosomes - macroparasites with an indirect life-cycle.. Proceedings of the Royal Society (London), Series B.

[B26] Gower CM, Shrivastava J, Lamberton PHL, Rollinson D, Emory A, Webster BL, Kabatereine NB, Webster JP (2006). Development and application of an ethical and epidemiologically appropriate assay for the multi-locus microsatellite analysis of  Schistosoma mansoni.. Parasitology.

[B27] Shrivastava J, Gower CM, Balolong E, Wang TP, Qian BZ, Webster JP (2005). Population genetics of multi-host parasites - the case for molecular epidemiological studies of Schistosoma japonicum using naturally sampled larval stages. Parasitology.

[B28] Morand S, Manning SD, Woolhouse MEJ (1996). Parasite-host coevolution and geographic patterns of parasite infectivity and host susceptibility. Proc R Soc Lond B.

[B29] Webster JP (2001). Compatibility and sex in a snail-schistosome system. Parasitology.

[B30] Richards CS (1970). Genetics of a molluscan vector of schistosomiasis. Nature.

[B31] Webster JP, Woolhouse MEJ (1999). Cost of Resistance: relationship between reduced fertility and increased resistance in a Schistosoma host-parasite system. Proceedings of the Royal Society, (London), Series B.

[B32] Webster JP, Davies CM, Hoffman JI, Ndamba J, Noble LR, Woolhouse MEJ (2001). Population genetics of Biomphalaria pfeifferi in the Zimbabwean highveld: implications for co-evolutionary theory.. Annals of Tropical Medicine and Parasitology.

[B33] Blair L, Webster JP (2006). Dose-dependent schistosome-induced mortality and morbidity risk elevates host reproductive effort. Journal of Evolutionary Biology.

[B34] Davies CM, Fairbrother E, Webster JP (2002). Mixed strain schistosome infections of snails and the evolution of virulence.. Parasitology.

[B35] Gower CM, Webster JP (2005). Intra-specific competition and the evolution of virulence in a parasitic trematode.. Evolution.

[B36] Sheldon BC, Verhulst S (1996). Ecological immunology: costly parasite defences and trade-offs in evolutionary ecology. Trends in Ecology and Evolution.

[B37] Morand S, Harvey P (2000). Mammalian metabolism, longevity and parasite species richness.. Proceedings of the Royal Society (London) Series B.

[B38] Moret Y, Schimd-Hempel P (2000). Survival for immunity: the price of immune system activation for bumblebee workers.. Science.

[B39] Taylor LH, Mackinnon MJ, Read AF (1998). Virulence of mixed-clone and single-clone infections of the rodent malaria Plasmodium chabaudi.. Evolution.

[B40] Woodruff DS, Cheng T (1985). Genetic control of schistosomiasis: a technique based on the manipulation of intermediate host snail populations. Parasitic and related diseases: Basic mechanisms, manifestations and control.

[B41] Hubendick B (1958). A possible method for schistosome-vector control by competition between resistant and susceptible strains. Bulletin of the World Health Organisation.

[B42] Fletcher M, Cheng T (1985). Genetic control of schistosomiasis: a mathematical approach.. Parasitic and related diseases: Basic mechanisms, manifestations and control.

[B43] Dieckmann U, Metz JAJ, Sabelis MW, Sigmund K (2002). Adaptive dynamics of infectious diseases: In Pursuit of Virulence Management.

[B44] Gilbert SC, Plebanski M, Gupta S, Morris J, Cox M, Aidoo M, Kwiatkowski D, Greenwood BM, Whittle HC, Hill AV (1998). Association of malaria parasite population structure, HLA, and immunological antagonism.. Science.

[B45] Veazey R, Ling B, Pandrea I, McClure H, Lackner A, Marx P (2003). Decreased CCR5 expression on CD4+ T cells of SIV-infected sooty mangabeys.. AIDS Research into Human Retroviruses.

[B46] Chen Z, Kwon D, Jin Z, Monard S, Telfer P, Jones MS, Lu CY, Aguilar RF, Ho DD, Marx PA (1998). Natural infection of a homozygous delta24 CCR5 red-capped mangabey with an R2b-tropic simian immunodeficiency virus.. Journal of experimental medicine.

[B47] Galvani AP, Novembre J (2005). The evolutionary history of the CCR5-Delta32 HIV-resistance mutation.. Microbes and Infection.

[B48] Kiepiela F, Leslie AJ, Honeyborne I, Ramduth D, Thobakgale F, Chetty S, Rathnavalu P, Moore C, J PK, Hilton L, Zimbwa P, Moore S, Allen T, Brander C, Addo MM, Altfeld M, James I, Mallal S, Bunce M, Barber LD, Szinger J, Day C, Klenerman P, Mullins JA, Korber B, Goovadia HM, Walker BD, Goulder PJ (2004). Dominant influence of HLA-B in mediating the potential coevolution of HIV and HLA.. Nature.

[B49] Blair L, Webster JP, Barker GC (2001). Isolation and characterization of polymorphic microsatellite markers in Schistosoma mansoni from Africa. Molecular Ecology Notes.

[B50] Jones CS, Lockyer AE, Rollinson D, Piertney SB, Noble LR (1999). Molecular Ecology.

[B51] Wiest PM (1996). The epidemiology of morbidity of schistosomiasis. Parasitology Today.

[B52] Newman JA, Bergelsen J, Grafen A (1997). Blocking factors and hypothesis tests in ecology: is your statistics text wrong ?. Ecology.

[B53] Perenger TV (1998). What's wrong with Bonferroni adjustments. British Medical Journal.

[B54] Garcia LV (2004). Escaping the Bonferroni claw in ecological studies. Oikos,.

[B55] Nakagawa S (2004). A farewell to Bonferroni: the problems of low statistical power and publication bias.. Behavioural Ecology.

[B56] Moran MD (2003). Arguments for rejecting the sequential Bonferroni in ecological studies. Oikos.

[B57] Schneider S, Roesli D, Excoffier L (2000). Arlequin.

[B58] Excoffier L, Smouse P, Quattro J (1992). Analysis of Molecular variance inferred from metric distances among haplotypes: Application to human mitochondrial DNA restriction data.. Genetics.

[B59] Wright S (1978). Evolution and Genetics of Population.

[B60] Mavarez J, Amarista M, Pointier JP, Jarne P (2000). Microsatellite variation in the freshwater schistosome-transmitting snail Biomphalaria glabrata. Molecular Ecology Notes.

